# Identifying critical components for railways rolling stock reliability: a case study for Iran

**DOI:** 10.1038/s41598-024-62841-2

**Published:** 2024-05-27

**Authors:** Seyed Farboud Seyedan Oskouei, Mehdi Abapour, Mojtaba Beiraghi

**Affiliations:** 1 Department of Electrical Engineering, Urmia Branch, Islamic Azad University, Urmia, Iran; 2 Department of Electrical and Computer Engineering, University of Tabriz, Tabriz, Iran

**Keywords:** Energy science and technology, Engineering, Mathematics and computing

## Abstract

Electrical railways constitute a vital component of transportation infrastructure worldwide, with rolling stock representing a key element of these systems. Given the extensive operational hours of such systems, effective maintenance scheduling and asset management are imperative to ensure reliability and safety while mitigating costs. This paper addresses the challenge of optimizing maintenance practices for railway rolling stock by introducing a novel implementation of reliability-centered maintenance (RCM) grounded in the reliability block diagram (RBD) framework. This methodology meticulously incorporates reliability parameters into maintenance strategies, aiming to enhance the operational efficiency of railway systems. Leveraging the criticality index, the study identifies components crucial for train reliability, facilitating cost-effective maintenance management. The proposed approach is applied and validated on the Tabriz line 1 metro in Iran, a system with over six years of operational history. Analysis reveals the bogie subsystem's criticality due to its interconnected components, with parts exhibiting significant mean time to repair (MTTR). Conversely, the brake system emerges as the most reliable subsystem. Additionally, sensitivity analysis demonstrates an inverse relationship between repair rates and component sensitivity, highlighting the pivotal role of efficient repair processes in bolstering system reliability. This research contributes a comprehensive and validated methodology for RCM in railway rolling stock, emphasizing cost reduction, system reliability, and strategic prioritization of maintenance efforts. As the approached method in this research is not limited to the specific case study and can be applied in any system by generating the RBD and reliability parameters of the system we want to study The findings hold significant implications for the global planning and execution of railway maintenance operations, setting a new standard for reliability-centered maintenance practices in the field.

## Introduction

### Motivation and background

Railway systems played an important role in all transportation systems such as passenger or freight. Electric railway systems are an important part of the urban transportation system in big cities. City managers must maintain this system with confidence because just one hour offline can cause chaos in city traffic. On the other hand, like all heavy industry systems, their maintenance costs are very high. The organization tries to reduce its maintenance costs and keep its system reliable. In the past decades, reducing maintenance costs and finding an optimal policy for maintenance has always been one of the most important concerns^1–5^. The high stakes in managing such complex systems require a strategic approach to maintenance and repair activities. Traditional methods often fail to address the delicate demands of modern electric railway systems, underscoring the need for a more rigorous and evidence-based approach. Therefore, it is necessary to use a method to reduce the costs of maintenance and repairs with timing, so that in addition to reducing costs, the reliability of the system is also maintained at a reasonable level. Therefore, it is necessary to reach a scientific and simple way to plan the maintenance and repairs of the railway fleet based on the reliability parameters of the fleet, so that by using it, it is possible to reach an optimal point of reliability and maintenance costs.

### Literature review and research gap

The most crucial parameter to choosing the right maintenance policy is the reliability index of the system, and many studies and methods based on reliability introduced in the last years^6–9^. Reliability-centered maintenance is one of them, the concept of RCM was developed in the early 1970s^10^, and it continued with different studies in the 80 s and 90s^11–13^ in this field. References^14–16^ are some guides and handbooks published for RCM. Nowadays, various engineering organizations use RCM for maintenance scheduling and asset management. In Ref.^17^, the most frequently used maintenance strategies are reviewed. Especially in electrical engineering, RCM is very famous, and much research has been done in the last few years. For distribution systems, a comprehensive scheme for reliability-centered maintenance is proposed in Refs.^18,19^, and approaches for identifying critical components for the reliability of distribution systems are presented in Refs.^20–22^. References^23,24^ compared the different maintenance strategies and policies in the distribution system and reported their effect on system reliability and cost. The impact of RCM in the maintenance optimization of a power distribution system is proposed in Refs.^25,26^. RCM benefits in the budgeting of maintenance programs are presented in Refs.^27–29^. For power planet, reference^30^ introduces a method to modeling combined cycle power plant and implantation RCM for reliability and availability analysis, critical components of combined cycle power plants Identified in Ref.^31^. In Refs.^32,33^, methods for identifying critical components for the reliability of transmission systems are proposed. RCM effect is also studied in transformers^34^ and electric ships^35^. It has also done some research to apply RCM methodology in the railway transportation system; reference^36^ introduced a maintenance management method in railway infrastructures like trucks and related equipment based on the reliability analysis and identifying the most critical items. In Refs.^37–41^, some strategies for choosing the optimal maintenance program and scheduling preventive maintenance activities in the railway system are proposed. Reference^42^ showed the impact of using new technologies in maintenance activity. An approach for modeling traction power supply systems (TPSS) is presented in Ref.^43^, and as illustrated in Ref.^44^, methods for maintenance decision optimization in TPSS can reduce system operating costs. RCM implanting on some subsystems of the railway rolling stock is introduced in Refs.^45,46^, reference^45^ selected metro doors system to applied RCM, and in Ref.^46^ wheelset of the train is the case study. In Ref.^47^, a study on the application of RCM is presented in metro railways, emphasizing its effectiveness in optimizing maintenance activities and enhancing system reliability. An approach to improving locomotive maintenance organization systems is suggested in Ref.^48^ through the introduction of RCM, highlighting its potential for enhancing maintenance efficiency and reducing costs. In Ref.^49^, a risk-based modeling approach is presented to optimize the maintenance of railway rolling stock, demonstrating the application of RCM principles in mitigating maintenance risks and improving reliability. In Ref.^50^, a reliability-based advanced maintenance modeling approach is developed for rolling stock manufacturers, showcasing how RCM can align maintenance activities with organizational objectives to enhance reliability and performance. In Ref.^51^ illustrates maintenance activities planning and grouping for complex structure systems and The maintenance scheduling within rolling stock planning, incorporating RCM principles is presented in Ref.^52^ to address uncertainties in maintenance durations and optimize maintenance schedules for improved reliability. The application of RCM in railway track maintenance is evaluated in Ref.^53^ highlighting its role in optimizing track maintenance strategies and improving overall system reliability. A new reliability-based model for optimizing stock maintenance of railroad passenger wagons is recommended in Ref.^54^, demonstrating the integration of RCM principles to enhance maintenance decision-making and reliability. The RCM methodology is implemented in Ref.^55^ indoor systems of train carriages, showcasing its effectiveness in optimizing door system maintenance activities and improving reliability. A practical hybrid model for optimizing the reliability, risk, and maintenance of rolling stock subsystems is demonstrated in Ref.^56^, illustrating how RCM principles can be integrated with risk assessment techniques to improve reliability and reference Ref.^57^ Showed the impact of maintenance strategies to control railway riskThe authors of the study in Ref.^58^ focus on improving the reliability of rolling stock door systems through maintenance optimization, highlighting the application of RCM principles in identifying and prioritizing maintenance tasks. A composite hybrid framework for optimizing the reliability of rolling stock subsystems is developed in Ref.^59^, showcasing the integration of RCM with other optimization techniques to improve overall system reliability. In Ref.^60^ a literature review on the cost of rolling stock maintenance in urban railway operations is conducted, emphasizing the importance of RCM in minimizing maintenance costs while ensuring reliable operation.

The exploration of existing scholarly work reveals a pronounced deficiency in the study of the RCM strategies specific to the railways rolling stock system. This shortfall points towards a compelling need for the development of an innovative, encompassing strategy for RCM application in railways rolling stock, one that meticulously outlines the system's critical and sensitive components through a detailed reliability model. Importantly, the methodology in question should possess sufficient adaptability to ensure its applicability across a broad spectrum of transportation systems, necessitating only minor modifications to parameters for its effective deployment in maintenance scheduling. This discernible gap in the research landscape underscores the absence of an optimized method for orchestrating the maintenance of Railways Rolling Stock that concurrently aims at cost minimization and the preservation of system reliability. Addressing this gap is paramount for enhancing the operational efficacy and dependability of Railways Rolling Stock, presenting a significant opportunity for future research endeavors in this domain.

### Paper contributions

In the railway industry, the RAMS documents as they are generated from the supplier they are focused just on one subsystem so don’t analyze the whole system and do not indicate each component how can be critical for the train it just guarantees the failure rate but This study introduces a groundbreaking methodology anchored in the Reliability Block Diagram (RBD) framework and Markov theory which they result are trusted in reliability evaluation, specifically tailored for the maintenance planning of railway rolling stock. This approach stands out by its meticulous consideration of reliability parameters in the formulation of maintenance strategies, aimed at enhancing the operational efficiency of railway systems. The core contributions of this research are multifaceted and significant in advancing the field of railway system maintenance as outlined below:

- *Innovative application of RBD in railways maintenance* At the heart of this study is the strategic application of the RBD framework, customized for the unique demands of railway rolling stock maintenance planning. This novel application not only illuminates the pathway to optimizing maintenance operations but also serves as a blueprint for integrating reliability parameters into maintenance planning processes systematically.
- *Cost reduction while maintaining system reliability* A pivotal achievement of this research is the demonstration of how strategic maintenance planning, guided by the proposed RBD-based method, can significantly reduce operational costs without compromising the reliability of the railway systems. This delicate balance between cost efficiency and operational reliability represents a substantial advancement in the maintenance planning domain. For example, spare part costs in the industry like railways are very expensive, and spare part management is very important for organizations, Based on this approach maintenance managers can realize which components are more critical for keeping the train in operation and manage their budget for arrange the warehouse and don’t waste budget on the not important part.
- *Optimization of reliability and maintenance costs* By employing the proposed method, this study successfully identifies the optimal point of intersection between reliability and maintenance costs. This optimization is crucial for railway operators seeking to maximize the cost-effectiveness of their maintenance expenditures while ensuring the uninterrupted reliability of their rolling stock they modify their preventive maintenance schedule based on the result.
- *Critical component index calculation* An analytical exploration into the reliability and availability of system equipment, as derived from the RBD model, facilitates the calculation of a critical index for system components. This index serves as a powerful tool for prioritizing maintenance efforts on components that are most critical to the system’s overall reliability and performance.
- *Real-world implementation and validation* The practical application and validation of the proposed method on Tabriz line 1 metro, located in the west of Iran, underscore its effectiveness and applicability in real-world settings. This implementation not only tests the method's viability but also showcases its potential to significantly enhance the maintenance operations of urban railway systems.

In essence, this study contributes a comprehensive and effectively validated methodology for RCM in railway rolling stock, emphasizing cost reduction, system reliability, and the strategic prioritization of maintenance efforts. The insights and outcomes from this research have the potential to significantly influence the planning and execution of maintenance operations in railway systems globally, setting a new benchmark for reliability-centered maintenance practices in the field, as this is important to practically analyze, test, and collect data for the proposed novel this research chose a metro system as the case study but the approached method can be successfully applied to any rolling stock even in the new or old systems it just makes a different in generating RBD and reliability analyzing.

### Paper structure

This paper is organized as follows: Sect. “Problem theoretical basis” briefly presents the theoretical basis of the proposed method for calculating different reliability parameters in an engineering system based on the Markov model. Deriving steps an RBD for electric railway rolling stock are presented in Sect. “Deriving steps of the electrical railway rolling stock RBD”. Section “Proposed reliability model of electrical railway’s rolling stock” introduced reliability modeling of an electrical railway's rolling stock and demonstrated the RBD of a sample system. The proposed approach is practically applied in an actual case study in Sect. “Practical case study”, and the result is presented. Finally, the conclusion is accomplished in Sect. “Conclusion”.

## Problem theoretical basis

The reliability assessment of electric railway rolling stock is a critical aspect of railway engineering and operations management, ensuring the safe, efficient, and timely movement of goods and passengers. Rolling stock refers to the vehicles that move on a railway, including locomotives, passenger cars, and freight wagons. Reliability, in this context, pertains to the ability of the rolling stock to perform its required functions under stated conditions for a specified period without failure. All engineering systems contain several subsystems connected in parallel or in a series arrangement. Electric trains as a system with repairable parts include different subsystems. e.g. the traction system, the brake system, the auxiliary system, and all of them have many components that are connected in parallel and series. For reliability analysis of the electrical railway's rolling stock, we must first reach the reliability modeling of the train, which identifies the different parts of a train and how to connect in a series and parallel arrangement. The system is studied in the lifetime period, the failure rate during this time is constant and the degradation and aging are not the content of this research, the calculation of different reliability parameters in an engineering system such as availability and unavailability indices, the system's mean time to failure, mean cycle time, and mean downtime and mean uptime will be described in this section^61,62^. Reliability and availability have different explanations in an engineering system. Availability shows the system probability that it is not under repair and not faulty when we need it to work well. Therefore, availability is related to reliability and maintainability. The reliability block diagram (RBD) of the electrical railway's rolling stock is being achieved and analyzed in the next section.

Reliability block diagram (RBD) extraction is a crucial process in the reliability assessment of electric railway rolling stock. RBD is a graphical representation of the components of a system and how they contribute to the overall system's reliability. RBD provides a systematic method to analyze the reliability of complex systems by breaking down the system into smaller, manageable blocks or components. This helps in identifying critical components that have a significant impact on the overall reliability. By visualizing the relationships and dependencies between different components, RBD helps in identifying weak points or vulnerabilities in the system that may lead to failures. This enables targeted improvements to enhance reliability. RBD can help in planning for redundancy and fail-safes by showing where parallel systems or backup components can be implemented to improve reliability and minimize downtime in case of a component failure. Through the analysis of RBD, maintenance teams can predict potential failures and plan maintenance activities more effectively. This predictive approach to maintenance ensures that components are serviced or replaced before they fail, thereby improving reliability. RBD extraction provides crucial data that supports decision-making processes regarding investments in maintenance, upgrades, and new acquisitions. It helps in prioritizing investments based on their potential impact on system reliability.

In summary, the reliability assessment of electric railway rolling stock, supported by RBD extraction, is fundamental to ensuring safety, efficiency, and customer satisfaction in railway operations. It provides a systematic approach to managing and improving the reliability of rolling stock, leading to better service delivery and operational performance.

### Reliability evaluation for a parallel arrangement system

**Figure 1:**
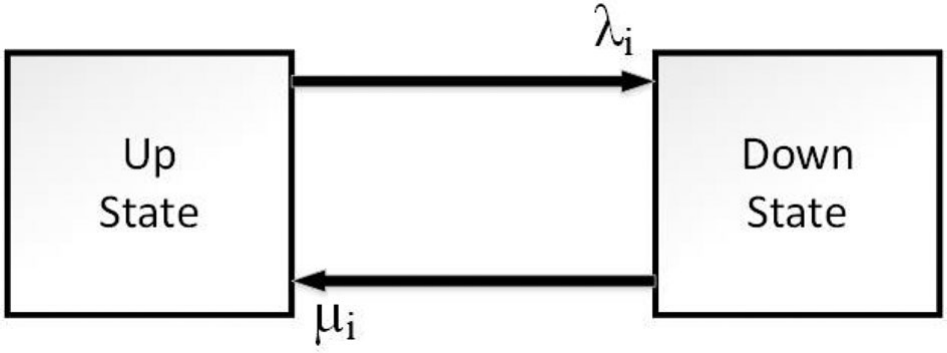
Electric train rolling stock Markov model with two state configurations.

Reliability evaluation in a series connection system when the components are repairable and independent is calculated in this section. Notice that all parts follow the Markov model with two state configurations shown in Fig. 1. This research base for the reliability model is failures that stop trains and cannot move after they happen so in this type of failure components completely lose their performance.

Equations (1) and (2) show how to reach the steady-state probabilities through part failure and success states and can calculate the repair rate with (3). Based on the concept of reliability in Ref.^61^, frequencies of the system in the steady-state situation can be obtained through (4). From (3) and (4), can reach the failure rate of the system under study via (5). In the end, the mean cycle time and mean downtime indices of the system will be obtained respectively through (6) and (7)^61^.

1 $$U = P_{F} \, , \, A = P_{S}$$

2 $$P_{F} = \prod\limits_{i \in N} {\left( {\frac{{\lambda_{i} }}{{\lambda_{i} + \mu_{i} }}} \right) \, } , \, P_{S} = 1 - \prod\limits_{i \in N} {\left( {\frac{{\lambda_{i} }}{{\lambda_{i} + \mu_{i} }}} \right)}$$

3 $$\mu_{S} = \sum\limits_{i \in N} {\mu_{i} } { , }f_{F} = f_{s} = P_{F} \mu_{s}$$

4 $$f_{S} = \left( {\prod\limits_{i \in N} {\frac{{\lambda_{i} }}{{\lambda_{i} + \mu_{i} }}} } \right).\left( {\sum\limits_{i \in N} {\mu_{i} } } \right),$$

5 $$\lambda_{S} = \frac{{f_{s} }}{{P_{S} }} = \frac{{\left( {\prod\limits_{i \in N} {\frac{{\lambda_{i} }}{{\lambda_{i} + \mu_{i} }}} } \right).\left( {\sum\limits_{i \in N} {\mu_{i} } } \right)}}{{\left( {1 - \prod\limits_{i \in N} {\frac{{\lambda_{i} }}{{\lambda_{i} + \mu_{i} }}} } \right)}},$$

6 $$MCT = \frac{1}{{f_{F} }} = \frac{1}{{\left( {\prod\limits_{i \in N} {\frac{{\lambda_{i} }}{{\lambda_{i} + \mu_{i} }}} } \right).\left( {\sum\limits_{i \in N} {\mu_{i} } } \right)}},$$

7 $$MDT = \frac{{P_{F} }}{{f_{F} }} = \frac{1}{{\mu_{S} }} = \frac{1}{{\sum\limits_{i \in N} {\mu_{i} } }}.$$

The MTTF indices show the mean time to failure, and the average elapsed time between inherent failures when the system is out of service. The MTTF is the same as MUT in a system with repairable parts obtained via (8) and (9).

8 $$MTTF = MUT{ , }MUT = MCT - MDT$$

9 $$MTTF = \frac{{\left( {1 - \prod\limits_{i \in N} {\frac{{\lambda_{i} }}{{\lambda_{i} + \mu_{i} }}} } \right)}}{{\left( {\prod\limits_{i \in N} {\frac{{\lambda_{i} }}{{\lambda_{i} + \mu_{i} }}} } \right).\left( {\sum\limits_{i \in N} {\mu_{i} } } \right)}}$$

The MTTFF represents the system's mean time to the first failure and can be calculated with the system transition rate matrix (10)^61^.

10 $$MTTFF = P_{ + } (0).( - R_{11} )^{ - 1} .U_{K}$$

$${\text{R = }}\left( {\begin{array}{*{20}c} {{\text{R}}_{{{11}}} } & {{\text{R}}_{{{12}}} } \\ {{\text{R}}_{{{21}}} } & {{\text{R}}_{{{22}}} } \\ \end{array} } \right)$$ is the system transition rate matrix, and ${\text{R}}_{{{11}}}$ showed the system transition rates from success to success. When all parts are upstate, ${\text{P}}_{ + } {(0)}$ represent the probability row vector for the system success states. The quantity of success states in the system is equal to the ${\text{U}}_{{\text{K}}}$^61^. Then suppose our system contains two repairable components with parallel connections. The MTTFF indices will be calculated via (11). The system components number in the parallel arrangement showed as the index.

11 $$MTTFF = \frac{{\lambda_{1} \left( {\lambda_{1} + \mu_{1} + \lambda_{2} + \mu_{2} } \right) + \mu_{1} \mu_{2} + \lambda_{2} (\mu_{1} + \lambda_{2} + \mu_{2} )}}{{\lambda_{1} \lambda_{2} (\lambda_{1} + \mu_{1} + \lambda_{2} + \mu_{2} )}}$$

### Reliability evaluation for a series arrangement system

Reliability evaluation in a series connection system, when the components are repairable and independent, is calculated in this section. It can reach the steady-state probabilities through part failure and success states (12) and (13). Equation (14) is used to calculate the system failure rate. Based on the reliability theory in Ref.^61^, steady-state frequencies of the system can be obtained through (15). Equations (13) and (15) can be used to reach the system-equivalent repair rate via (16). In the end, The MCT and MDT indices will be obtained through (17) and (18), respectively^61^. For a System with series-connected parts, MTTF and MTTFF are respectively expressed in (8) and (19), and for this kind of system, MTTF and MTTFF are equal. Other assumptions are the same as the parallel arrangement.

12 $$P_{F} = 1 - \prod\limits_{i \in N} {\left( {\frac{{\mu_{i} }}{{\lambda_{i} + \mu_{i} }}} \right)} { , }P_{S} = \prod\limits_{i \in N} {\left( {\frac{{\mu_{i} }}{{\lambda_{i} + \mu_{i} }}} \right)}$$

13 $$P_{F} = U = 1 - P_{S} = 1 - \prod\limits_{i \in N} {\left( {\frac{{\mu_{i} }}{{\lambda_{i} + \mu_{i} }}} \right)}$$

14 $$\lambda_{S} = \sum\limits_{i \in N} {\lambda_{i} } { , , }f_{F} = f_{s} = P_{S} \lambda_{s}$$

15 $$f_{F} = \left( {\prod\limits_{i \in N} {\frac{{\mu_{i} }}{{\lambda_{i} + \mu_{i} }}} } \right).\left( {\sum\limits_{i \in N} {\lambda_{i} } } \right),$$

16 $$\mu_{S} = \frac{{f_{F} }}{{P_{F} }} = \frac{{\left( {\prod\limits_{i \in N} {\frac{{\mu_{i} }}{{\lambda_{i} + \mu_{i} }}} } \right).\left( {\sum\limits_{i \in N} {\lambda_{i} } } \right)}}{{\left( {1 - \prod\limits_{i \in N} {\frac{{\mu_{i} }}{{\lambda_{i} + \mu_{i} }}} } \right)}},$$

17 $$MCT = \frac{1}{{f_{F} }} = \frac{1}{{\left( {\prod\limits_{i \in N} {\frac{{\mu_{i} }}{{\lambda_{i} + \mu_{i} }}} } \right).\left( {\sum\limits_{i \in N} {\lambda_{i} } } \right)}},$$

18 $$MDT = \frac{{P_{F} }}{{f_{F} }} = \frac{1}{{\mu_{S} }} = \frac{{\left( {1 - \prod\limits_{i \in N} {\frac{{\mu_{i} }}{{\lambda_{i} + \mu_{i} }}} } \right)}}{{\left( {\prod\limits_{i \in N} {\frac{{\mu_{i} }}{{\lambda_{i} + \mu_{i} }}} } \right)\left( {\sum\limits_{i \in N} {\lambda_{i} } } \right)}},$$

19 $$MTTFF = MTTF = \frac{1}{{\sum\limits_{i \in N} {\lambda_{i} } }}.$$

## Deriving steps of the electrical railway rolling stock RBD

**Figure 2:**
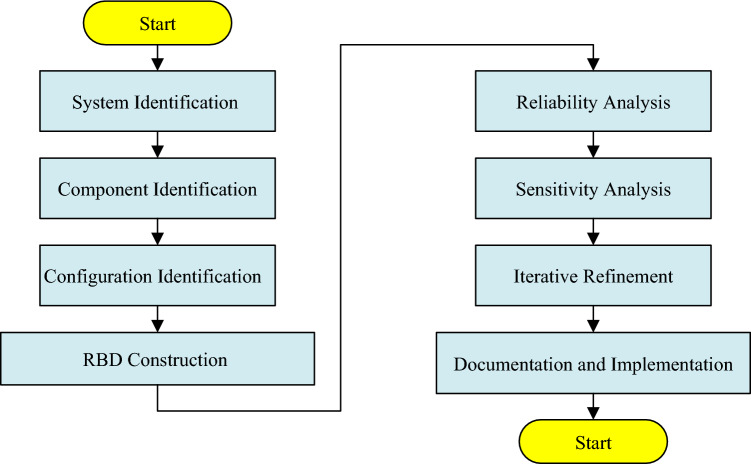
Steps of driving the RBD for electric railway rolling stock.

Generating RBD the system for analysis is a familiar method in engineering^63^ and also some studies Were done in railways system^64,65^. Deriving steps for an RBD for electric railway rolling stock involves a systematic approach to model the reliability and performance of the system based on its components and their interconnections are demonstrated in Fig. 2 and The process entails understanding the functional relationships between the components of the rolling stock, categorizing them into series and parallel configurations based on their operational dependencies, and analyzing how each component's reliability contributes to the overall system reliability. The classification used in this research is a regular classification in the railway industry and related to the case study but it can be changed depending on our purpose. Here's how to derive an RBD for electric railway rolling stock:

### Step 1 System identification

#### Define the system boundaries

Delineate the scope of the rolling stock system, including all relevant components such as propulsion systems, braking systems, power supply, control systems, and safety systems. Classification of the system in some subsystems helps to study more detail but it will not impact the final result because each component independently affects reliability analysis, we can study the system as one part or separate that in some subsystems.

#### Identify key functions

List the critical functions necessary for the operational reliability of the rolling stock, such as traction, control, braking, and passenger safety.

### Step 2 Component identification

#### List components

Identify all components and subsystems involved in each key function of the rolling stock. This includes engines, motors, brakes, electronic control units, power converters, couplers, and onboard safety devices.

#### Understand component relationships

Determine how components interact and depend on each other for the rolling stock to function reliably.

### Step 3 Configuration identification

#### Series configuration

Components in a series configuration must all function for the system to work. Failure of any single component leads to system failure. This is common in critical paths where the operation depends on the sequential reliability of components.

#### Parallel configuration

Components in a parallel configuration increase system reliability since the system can continue to function if one component fails. This is often used for redundancy in safety–critical systems.

### Step 4 RBD construction

#### Map components to RBD

Draw the RBD, placing components according to their operational dependencies (series or parallel).

#### Incorporate failure and repair rates

For each component, include relevant data on failure rates (λ) and, if applicable, repair rates (μ) to model the system’s reliability over time.

### Step 5 Reliability analysis

#### Calculate component reliability

Use failure rate data to calculate the reliability of individual components over the desired period.

#### Determine system reliability

Apply reliability formulas for series and parallel configurations to calculate the overall system reliability. For series systems, the overall reliability is the product of the reliabilities of individual components. For parallel systems, the calculation is more complex, often involving the complement of the product of unreliabilities.

### Step 6 Sensitivity analysis

#### Identify critical components

Use the RBD to identify components whose failure significantly impacts system reliability. These are candidates for improved maintenance or redesign for higher reliability.

### Step 7 Iterative refinement

#### Review and update RBD

As components are upgraded or system configurations change, periodically review and update the RBD to reflect these changes and their impact on system reliability.

### Step 8 Documentation and implementation

#### Document RBD and findings

Document the RBD, including assumptions, component reliability data, and system reliability calculations.

#### Implement reliability improvements

Based on the RBD analysis, implement improvements to the rolling stock's design, maintenance, and operational procedures to enhance reliability.

Deriving an RBD for electric railway rolling stock is a complex but critical process that helps in understanding and improving the reliability of the system. It requires a thorough understanding of the system's components, their failure modes, and how they interact within the system to deliver reliable performance.

## Proposed reliability model of electrical railway’s rolling stock

### The proposed RBD

**Figure 3:**
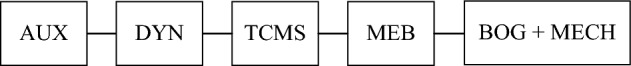
The RBD of the electrical railway’s rolling stock with different sub-system.

**Figure 4:**
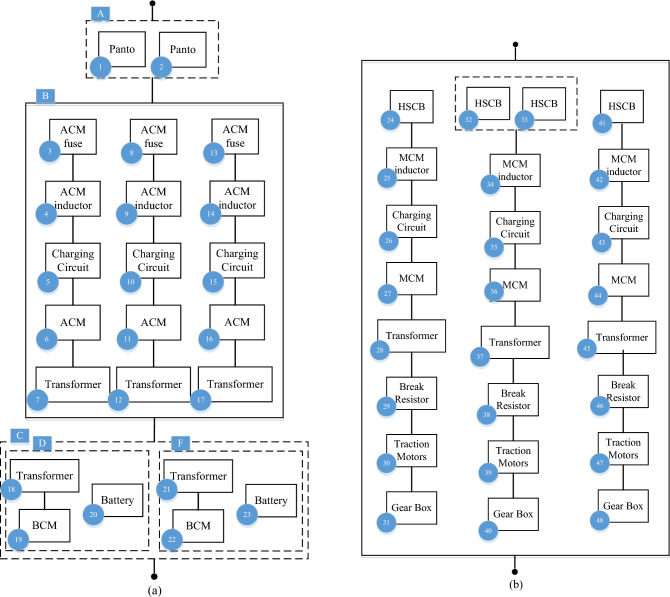

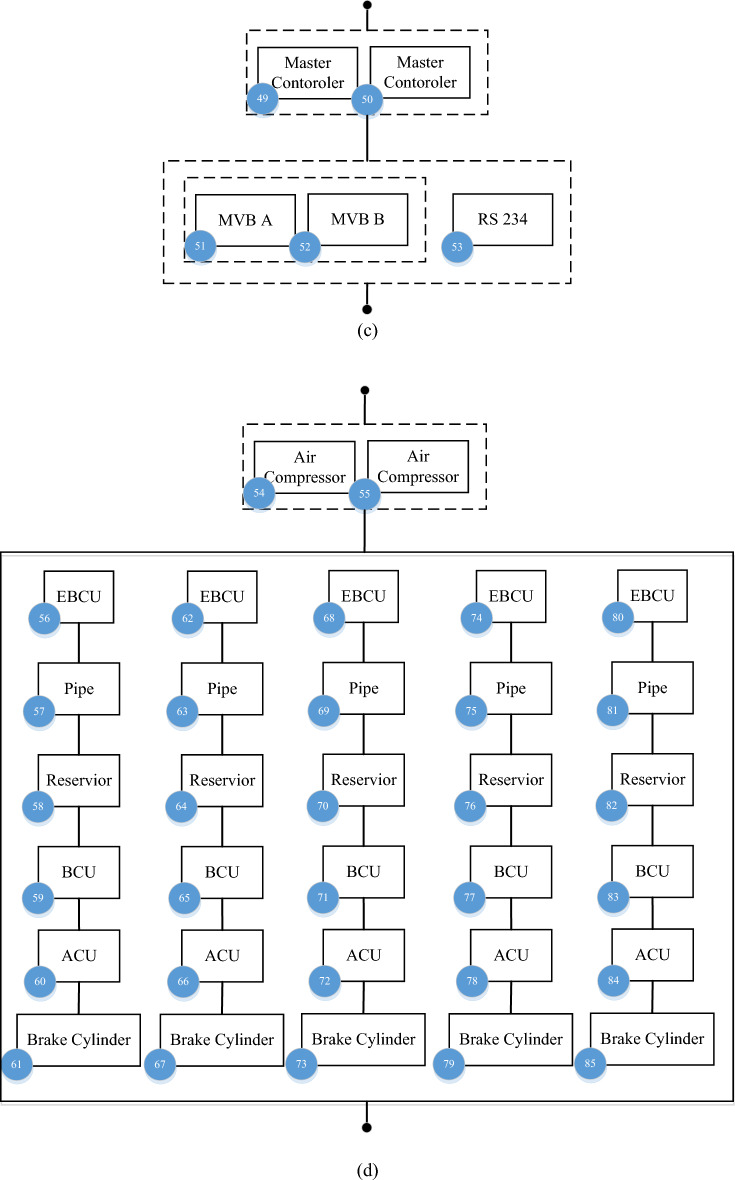

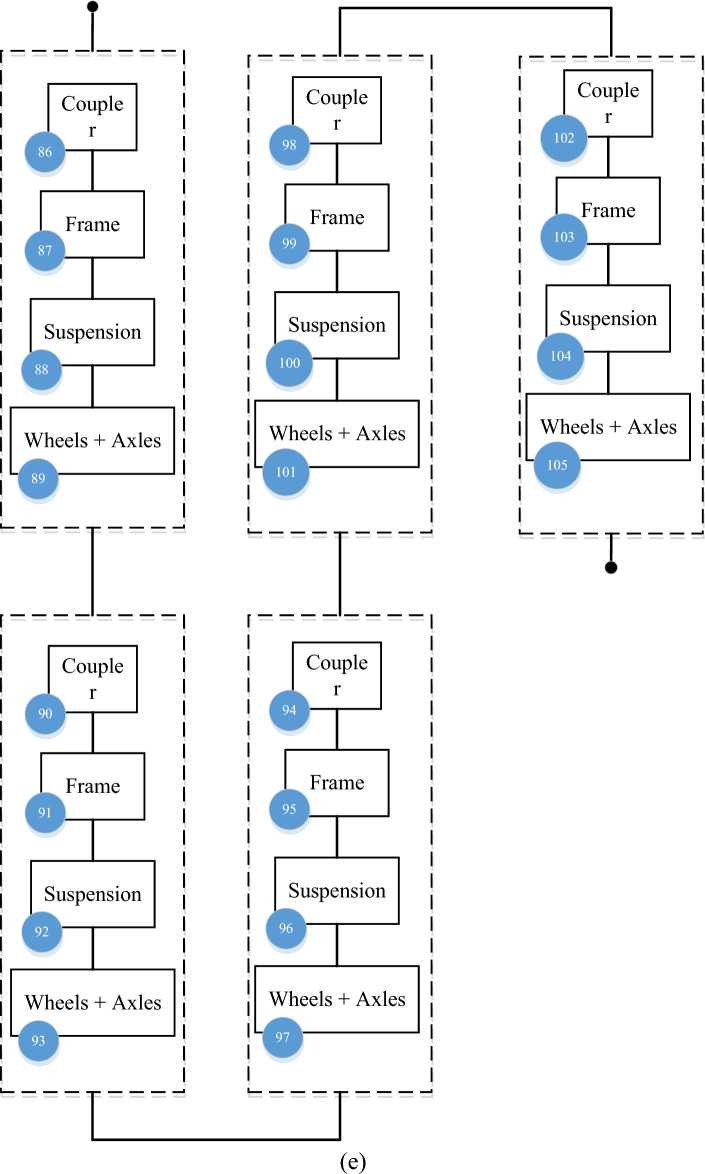
Different component of the electrical railway’s rolling stock RBD sub-systems (**a**) AUX (**b**) DYN (**c**) TCMS (**d**) MEB (**e**) BOG.

This day’s electrical railways contain different perspectives trying to solve any transportation need. Some varieties are used in cities like metro, tram, or monorail, and some are used in mainline such as high-speed trains, and regional or electric locomotives to transport people and cargo all around the country. This section will present a new reliability model for the metro type, the most popular electric railway type. The reliability model depends on what kind of failures we are studying, e.g., some of them can cause a delay in the operation or can stop the train and will be needed to rescue. Our research base for the reliability model is failures that stop trains and cannot move after they happen for example one coupler or axle broken or losing both pantographs and failures that cause delays in the operation timetable or reduce component performance are not considered like faults in one door or saloon lighting. The RBD of the electrical railway’s rolling stock with different sub-systems is demonstrated in Fig. 3. For exporting the RBD, a comprehensive study of technical documents, available diagrams, and discussions with maintenance engineers in Tabriz metro line 1 in the west of Iran were done. The reliability model proposed for this rolling stock contains six main subsystems: Auxiliary power supply (AUX), Traction (DYN), Train control and management system (TCMS), Mechanical braking (MEB), Bogie (BOG), and Vehicles coupling (MECH). Each subsystem has several parts with different functions that connect and work well. Different components of the electrical railway’s rolling stock RBD sub-systems such as AUX, DYN, TCMS, MEB, and BOG are depicted in Fig. 4. To reach the RBD of the case study this research used expert maintenance engineers who dominate the case studys rolling stock design.

### The electrical railway’s rolling stock RBD sub-systems

In the following, different components of the electrical railway’s rolling stock RBD sub-systems such as AUX, DYN, TCMS, MEB, and BOG are based on the case study are introduced.

#### Auxiliary power supply (AUX)

AUX is a power supply system for the auxiliary equipment of the train, where the received power of 1500 V DC enters this system through pantographs and is converted into three-phase power of 400 V AC to provide the power required by systems such as air conditioning, lighting, and cooling fans. This subsystem has a main inverter called an auxiliary converter module (ACM) which converts electricity in this equipment. Also, a DC/DC converter named battery converter module (BCM) is installed in this subsystem to provide the 110-V DC power required by the control circuits and charge the train batteries until the pantograph is not connected to the network and guarantee the continuous operation of control systems and emergency lighting. In addition, Panto is a device for transferring electricity from the main power grid to the train. AUX subsystem includes equipment such as circuits and filters to protect the main transformer of the subsystem (Charging Circuit, ACM fuse, and ACM inductor). Each of the main converters and battery charger has a transformer. The battery also guarantees the continuous operation of critical systems in emergencies and lack of mains electricity. If the train loses the first ACM, the TCMS system cuts the air condition compressor power and if the train loses the second ACM TCMS system will cut also the normal ventilation and normal light train uses 110DC to start emergency ventilation and keeps emergency light and control system, so just one ACM is enough for the train to continue moving but it does mean training keep 100% performance, some of the unnecessary demand will be cut off.

#### Traction (DYN)

DYN is a power supply system for creating driving force and thrust of the train, which converts the received 1500 V DC power into 1400 V AC three-phase power to provide the power required for the movement of 12 250 KW train motors. This sub-system has a main inverter named motor converter module (MCM) and electricity conversion is done in this equipment. The converted electricity is transferred to the electric motors so that the driving force is transferred to the wheels through the gearbox. The components used in this subsystem include high-speed circuit break (HSCB), MCM inductor, charging circuit, Transformer, Brake resistor, and Gearbox. HSCB is high-pressure and high-speed switches installed in the power supply path of the train traction system. MCM inductor and charging circuit is a group of circuits and filters to protect the main converter, that is, MCM, and the main converters have a transformer. The brake resistor is used to protect the converter from increasing the network voltage as well as the return braking energy loss, and the gearbox is applied for the mechanical transmission of the driving force produced in the engines to the wheels. If the train loses the first MCM, TCMS shows an event to the driver and the train keeps moving normally but if the train loses the second MCM train will be stopped and for moving the train driver must be switched to the emergency traction mode then can be a movie with limited speed. In this case study the train is designed to rescue another train with full passengers.

#### Train control and management system (TCMS)

TCMS is a train control and management system, where decisions and orders are analyzed in this subsystem. This subsystem includes the master controller, MVB, and RS234 components. Motion commands such as traction and braking are sent by the driver with the master controller. MVB and RS234 are infrastructures of communication network of components in the TCMS subsystem and information exchange is done in these platforms.

#### Mechanical braking (MEB)

The MEB sub-system includes the train brake component and its accessories that work with compressed air pressure. This sub-system includes an electrical brake control unit (EBCU), air compressor, pipe, reservoir, auxiliary control unit (ACU), brake control unit (BCU), and Brake cylinder. EBCU is responsible for making decisions on valve operation and brake pressure regulation, air compressor is the compressed air supply unit for the brake system, Pipe is the piping system and compressed air transmission to the brake cylinders, Reservoir refers to the production of compressed air storage tanks. In compressors, ACU and BCU are a group of electric and pneumatic valves to execute the form issued by EBCU, and Brake cylinders are brake cylinders that the compressed air entering causes friction in the wheels and braking and stopping the train. In this case study the rain is designed to rescue another train with full passengers so brake calculation to keep the moving train when one car brake system works normally but in this kind of situation, TCMS order speed limitation.

#### Bogie, and vehicles coupling (BOG + MECH)

BOG + MECH subsystem is a group of main mechanical parts and components of the train including coupler, frame, suspension, and Wheels + Axles. The coupler component creates a connection between train cars. The frame is the body of the wagon to which other equipment is connected and accommodates passengers. Suspension is the suspension system of the train, which enables the smooth movement of the train and the comfort of the passenger. Wheels + Axles are the wheels and axles that the driving force transmitted through the engine rotates and leads to the movement of the train.

The Number of components used in different subsystems of the electrical railway’s rolling stock is given in Table 1.

**Table 1:** Number of components used in the electrical railway’s rolling stock.

Subsystem	Component	Number of components
AUX	Panto, ACM	1–23
DYN	Gear box, traction motors	24–48
TCMS	Master controller, MVB	49–53
MEB	Air compressor, pipe	54–85
BOG	Frame, wheels + Axles	86–105

### Reliability parameters

A major fault in the components can prevent the subsystems out of service and the train stopped from moving. The detailed reliability model of each subsystem is illustrated in Fig. 3a–e. For calculating reliability, failure data of the components will be used as an input to the reliability model and assumed the subsystems are independent. Applying the Eqs. (1)–(19) on the RBD can analyze the reliability of rolling stock.

The reliability parameters of the AUX subsystem as a repairable system are calculated, for example, the block A indices obtained through (20) and (21).

20 $$P_{A,F} = \frac{{\lambda_{1} \lambda_{2} }}{{\left( {\lambda_{1} + \mu_{1} } \right)\left( {\lambda_{2} + \mu_{2} } \right)}}, \, P_{A,F} = 1 - P_{A,F}$$

21 $$\mu_{A} = \mu_{1} + \mu_{2} , \, \lambda_{A} = \frac{{\lambda_{1} \lambda_{2} \left( {\mu_{1} + \mu_{2} } \right)}}{{\left( {\lambda_{1} + \mu_{1} } \right)\left( {\lambda_{2} + \mu_{2} } \right) - \lambda_{1} \lambda_{2} }}$$

After calculating block B and C parameters as a parallel system like block A, the reliability parameters of the AUX subsystem are expressed in (22)–(27):

22 $$A = \frac{{\mu_{A} \mu_{B} \mu_{C} }}{{\left( {\lambda_{A} + \mu_{A} } \right)\left( {\lambda_{B} + \mu_{B} } \right)\left( {\lambda_{C} + \mu_{C} } \right)}},$$

23 $$\lambda_{S} = \lambda_{A} + \lambda_{B} + \lambda_{C} ,$$

24 $$\mu_{S} = \frac{{\mu_{A} \mu_{B} \mu_{C} \left( {\lambda_{A} + \lambda_{B} + \lambda_{C} } \right)}}{{\left( {\lambda_{A} + \mu_{A} } \right)\left( {\lambda_{B} + \mu_{B} } \right)\left( {\lambda_{C} + \mu_{C} } \right) - \mu_{A} \mu_{B} \mu_{C} }},$$

25 $$MCT = \frac{{\left( {\lambda_{A} + \mu_{A} } \right)\left( {\lambda_{B} + \mu_{B} } \right)\left( {\lambda_{C} + \mu_{C} } \right)}}{{\mu_{A} \mu_{B} \mu_{C} \left( {\lambda_{A} + \lambda_{B} + \lambda_{C} } \right)}},$$

26 $$MDT = \frac{{\left( {\lambda_{A} + \mu_{A} } \right)\left( {\lambda_{B} + \mu_{B} } \right)\left( {\lambda_{C} + \mu_{C} } \right) - \mu_{A} \mu_{B} \mu_{C} }}{{\mu_{A} \mu_{B} \mu_{C} \left( {\lambda_{A} + \lambda_{B} + \lambda_{C} } \right)}},$$

27 $$MTTF = (\lambda_{S} )^{ - 1} = \left( {\lambda_{A} + \lambda_{B} + \lambda_{C} } \right)^{ - 1} , \, MTTFF{ = }MTTF$$

### The components criticality evaluation

For maintenance management and analyzing which parts play more critical roles in the train's reliability, it should apply the criticality evaluation to the system. Different methods are suggested for criticality evaluation, e.g. risk reduction worth^66^, Fussell–Vesely's method^67^, or Birnbaum's method^68^. This paper used the technique illustrated in (28)–(30) to identify the criticality index of components in the reliability or availability of the system^30^.

28 $$C_{{\lambda_{i} }}^{{\lambda_{sys} }} = - \left( {\frac{{\partial \lambda_{sys} }}{{\partial \lambda_{i} }}} \right)\Delta \lambda_{i} , \, C_{{\mu_{i} }}^{{\lambda_{sys} }} = - \left( {\frac{{\partial \lambda_{sys} }}{{\partial \mu_{i} }}} \right)\Delta \mu_{i}$$

29 $$C_{{\lambda_{i} }}^{{\mu_{sys} }} = - \left( {\frac{{\partial \mu_{sys} }}{{\partial \lambda_{i} }}} \right)\Delta \lambda_{i} , \, C_{{\mu_{i} }}^{{\mu_{sys} }} = - \left( {\frac{{\partial \mu_{sys} }}{{\partial \mu_{i} }}} \right)\Delta \mu_{i}$$

30 $$C_{{\lambda_{i} }}^{{U_{sys} }} = - \left( {\frac{{\partial U_{sys} }}{{\partial \lambda_{i} }}} \right)\Delta \lambda_{i} , \, C_{{\mu_{i} }}^{{U_{sys} }} = - \left( {\frac{{\partial U_{sys} }}{{\partial \mu_{i} }}} \right)\Delta \mu_{i}$$

Using of Eqs. (28)–(30), criticality index with different influence parameters can be measured. It is essential to know that the $\Delta \lambda_{i}$ and $\Delta \mu_{i}$ are two independent parameters. It means another one does not reflect any changes in one of them. The negative value of this two-parameter can be caused by reducing $\lambda_{i}$ or $\mu_{i}$.

The introduced approach can be applied entirely in other railway systems or any engineering systems with minimum change in the parameter. It just needed to reach the RBD of the case study in the first step and, after that, calculate the reliability parameter. This approach is instrumental in optimizing maintenance costs and Identifying critical components, which is essential to keep the system reliable.

## Practical case study

### General speciation of case study

**Figure 5:**
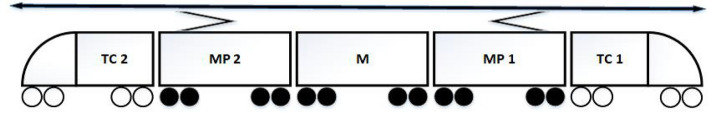
Tabriz metro line 1 trains combination.

For maintenance management and analyzing which parts play more critical roles in the reliability of the train, the criticality index is used. The railway system that is used to study in this paper is Tabriz Line 1 metro, located in the northwest of Iran and under operation and service for more than 6 years, Fig. 5 shows the studied train car combination, and the general speciations are as follows:

- Rolling stocks of Tabriz line 1 metro contains five cars which are named TC1, MP1, M, MP2, TC2,
- TC1 and TC2 are the trailer cars, including the driver's cabin.
- MP1, M, and MP2 are the trail-motored cars.
- 1500 DC power from the overhead catenary is transferred with two pantographs located in MP1 and MP2 to the train high voltage system and then distributed between 5 cars.
- Each motorized car contains four 250 KW motors.
- The train contains three motor converter modules (MCM) located in MP1, M, and MP2. The MCMs convert 1500 DC to 1400 AC (3ph) to supply the motors.
- The train contains three Auxiliary converter modules (ACM) located in TC1, M, and TC2. The MCM converts 1500 DC to 400 AC (3ph) to supply train auxiliary demands like light and air conditioning. The ACM also delivers needed power to the battery converter module (BCM) to provide the 110 DC demand-like control system.

### Critically indices results

Eight trains followed for more than four years to collect the reliability parameters of the case study of this paper. All kinds of failures listed, including the reason and the influence of that failure in the train and continuing the operation, also used Tabriz metro maintenance experts' experience to reach the reliability parameters. Reliability data related to AUX, DYN, TCMS, MEB, and BOG components are presented in Tables 2, 3, 4, 5 and 6, respectively.

**Table 2:** Failure and repair rates of AUX components.

Component	λ (f/y)	μ (r/y)
Panto	18.466074	1095
ACM fuse	2.603106	8760
ACM inductor	1.760022	1752
Charging circuit	2.009106	2925.84
ACM	2.999106	547.5
Transformer	4.115232	547.5
Transformer (BCM)	4.845258	547.5
BCM	3.390552	547.5
Battery	25.438644	4380

**Table 3:** Failure and repair rates of DYN components.

Component	λ (f/y)	μ (r/y)
HSCB	5.57271	2190
MCM inductor	1.876644	1752
Charging circuit	2.009106	2925.84
MCM	3.26205	547.5
Transformer	4.992372	547.5
Break resistor	5.790312	1095
Traction motors	6.365106	219
Gear box	2.030292	156.366

**Table 4:** Failure and repair rates of TCMS components.

Component	λ (f/y)	μ (r/y)
Master controller	26.6112	1752
MVB A	0.042372	876
MVB B	0.042372	876
RS 234	0.083754	1095

**Table 5:** Failure and repair rates of MEB components.

Component	λ (f/y)	μ (r/y)
Air compressor	1.103058	365.292
EBCU	0.442728	4380
Pipe	0.172458	365.292
Reservior	0.091674	2190
BCU	0.299178	547.5
ACU	0.268488	547.5
Brake cylinder	0.717354	876

**Table 6:** Failure and repair rates of BOG + MECH components.

Component	λ (f/y)	μ (r/y)
Coupler	0.223146	547.5
Frame	0.102564	109.5
Suspension	1.563804	365.292
Wheels + Axles	0.30987	219

**Figure 6:**
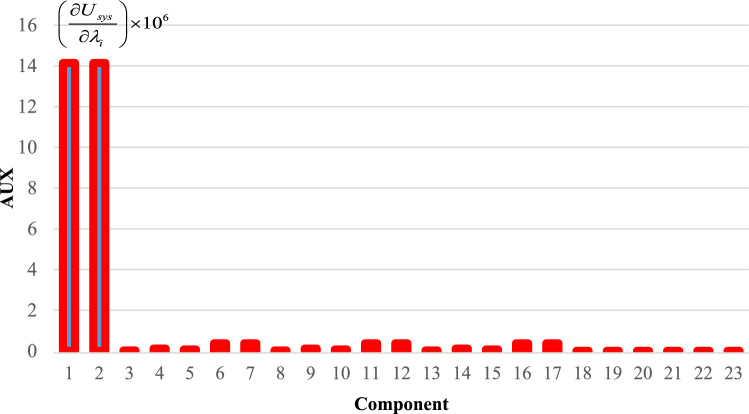
Critically indices of AUX components.

**Figure 7:**
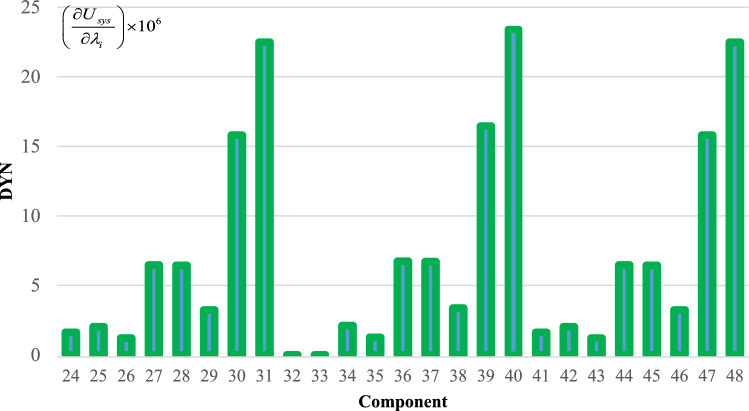
Critically indices of DYN components.

**Figure 8:**
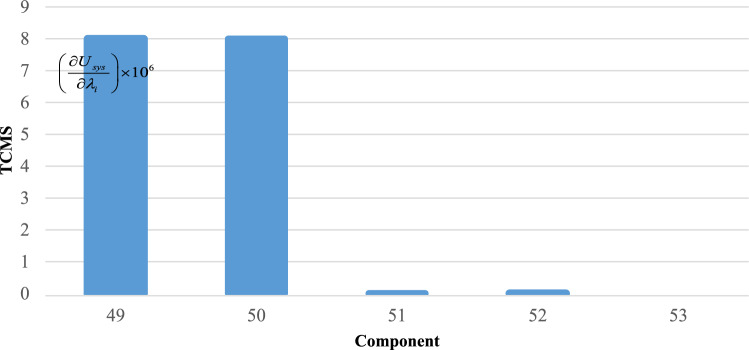
Critically indices of TCMS components.

**Figure 9:**
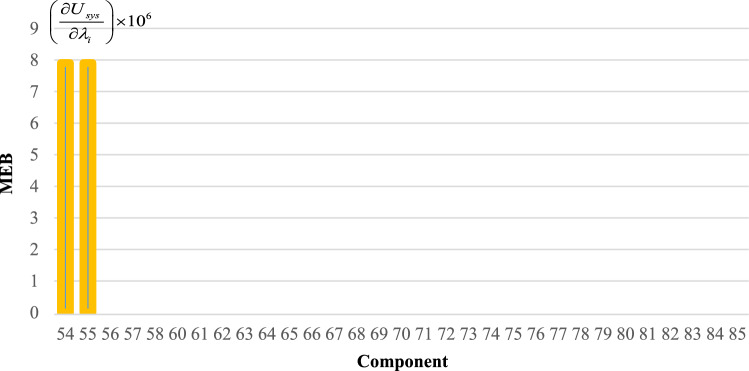
Critically indices of MEB components.

**Figure 10:**
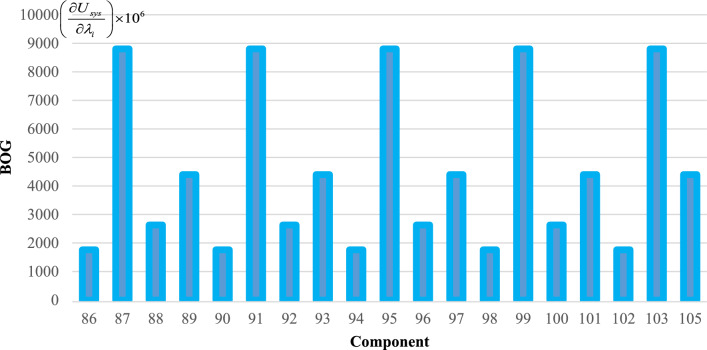
Critically indices of BOG components.

To effectively manage maintenance and identify key components affecting reliability, it is necessary to evaluate the sensitivity of the system. The proposed method aims to identify the critical components necessary to improve the reliability of trains. The indices $C_{{\lambda_{i} }}^{{U_{sys} }}$ for all components are obtained and demonstrated in Figs. 6, 7, 8, 9 and 10. For a better comparison, the sensitivity of each subsystem component is shown separately in Figs. 6, 7, 8, 9 and 10. The obtained results showed that the bogie subsystem has a higher sensitivity due to its interconnected parts, which show a significant MTTR. On the contrary, the results show that the braking system is the most reliable subsystem in the train. Therefore, the most important part in train reliability is related to the bogie subsystem because all components are connected in series.

Table 7, lists the critical components of each subsystem. The operating company can schedule maintenance programs based on this table data to keep the train much safer.

**Table 7:** Critical components of each sub-system.

Subsystem	Component	λ (f/y)	μ (r/y)	Critically indices
AUX	Panto	18.466074	1095	14.13452418
	ACM	2.999106	547.5	0.3795880416
DYN	Gear box	2.030292	156.366	23.41619361
	Traction motors	6.365106	219	16.45790826
TCMS	Master controller	26.6112	1752	7.995704357
	MVB	0.042372	876	0.000004073
MEB	Air compressor	1.103058	365.292	7.903102775
	Pipe	0.172458	365.292	0.000000097
BOG	Frame	1.563804	365.292	8802.039054
	Wheels + Axles	0.30987	219	4398.917608

### Sensitivity analysis

The analytical exploration presented in this study focuses on evaluating the reliability and availability of system equipment within railway rolling stock systems through a meticulously derived model. By calculating the critical index for the equipment, the research delves into the nuanced dynamics of maintenance priorities and operational readiness. In this section, a sensitivity analysis was performed based on Table 7 for the changes in the repair rate obtained, and the effect of increasing and decreasing changes of 50% on the sensitivity of the AUX, DYN, TCMS, MEB, and BOG components is evaluated.

**Figure 11:**
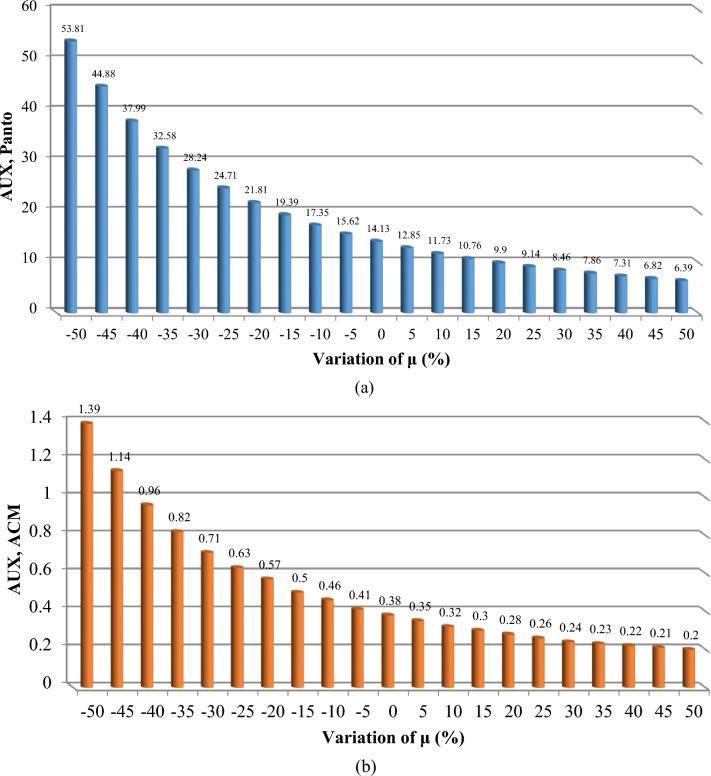
Effect of $\mu$ variations on AUX critically indices (**a**) Panto (**b**) ACM.

**Figure 12:**
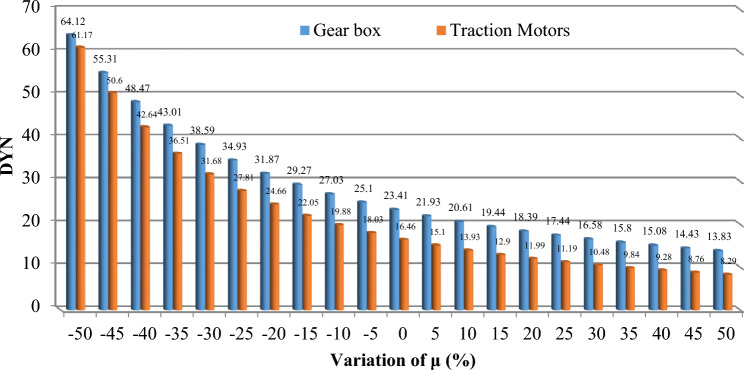
Effect of $\mu$ variations on DYN critically indices.

**Figure 13:**
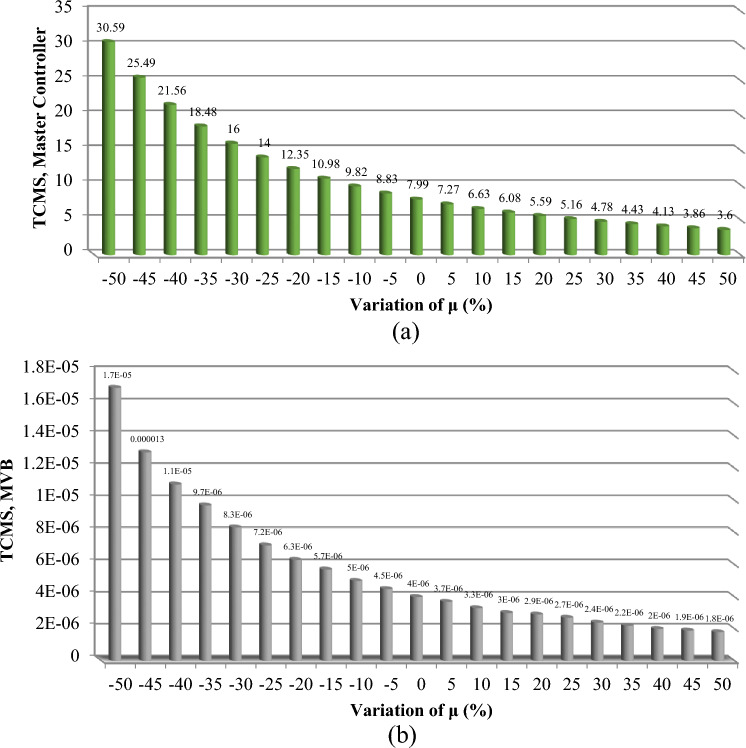
Effect of $\mu$ variations on TCMS critically indices (**a**) Master controller (**b**) MVB.

**Figure 14:**
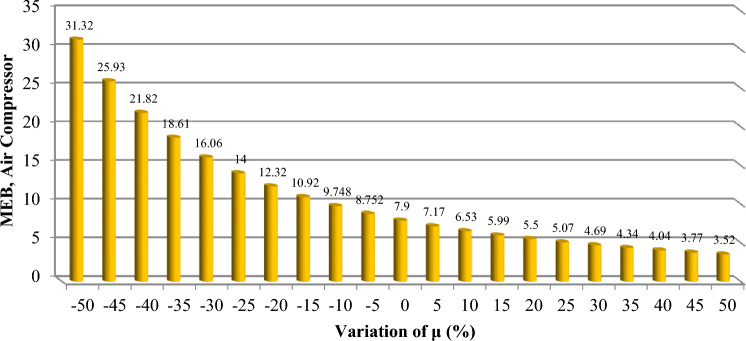
Effect of $\mu$ variations on MEB, critically index.

**Figure 15:**
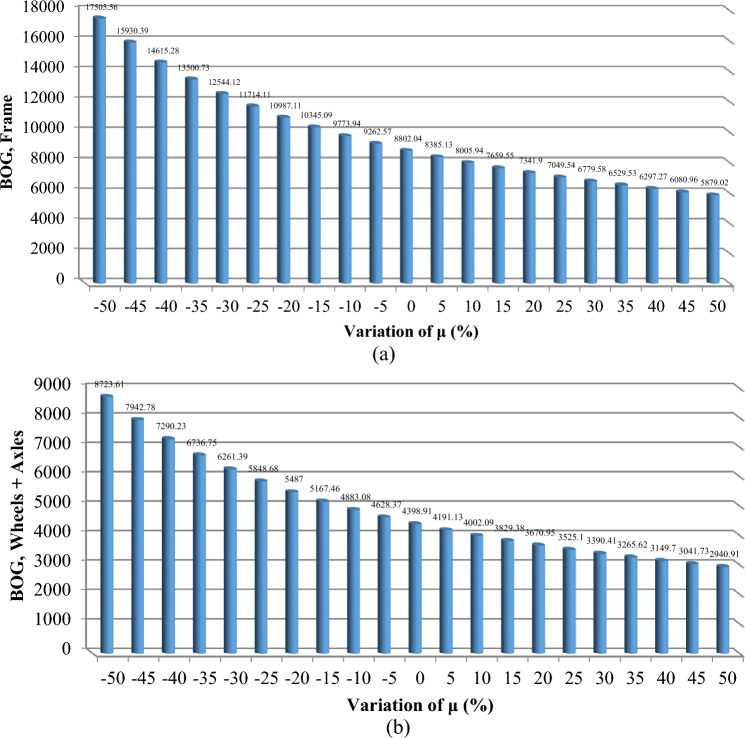
Effect of $\mu$ variations on BOG critically indices (**a**) frame controller (**b**) Wheels + Axless.

The findings, illustrated across Figs. 11, 12, 13, 14 and 15, offer a compelling insight into the relationship between repair rates and component sensitivity. It emerges that an increase in repair rates correlates with a decrease in sensitivity for the examined components. This inverse relationship underscores the critical role that efficient repair processes play in enhancing the robustness and reliability of railway rolling stock systems. Conversely, a decrease in repair rates heightens sensitivity, suggesting a heightened risk of system unreliability and potential operational challenges. This analysis not only highlights the importance of optimizing repair rates to balance system sensitivity and reliability but also sets a precedent for future research in maintenance strategy optimization. By identifying how variations in repair rates affect component sensitivity, the study provides a valuable framework for railway system operators to strategize maintenance interventions, thereby ensuring that the rolling stock remains reliable, available, and operationally efficient. MTTR is just only parameter that can be changed by the organizations because other parameters are related to the essence of the system, so improvements in human resource knowledge and equipment of repair salons can improve MTTR reduce cost, and make the train more reliable.

Based on Table 8, the evaluation of the sensitivity analysis performed for the effect of changes in equipment repair rates on their sensitivity has shown. The results showed that MVB, air compressor, master controller, panto and ECM equipment are more sensitive for 1% change in repair rate compared to other equipment and showed more changes.

**Table 8:** Percentage of critical components variation for each 1% repair rate change.

Subsystem	Component	Average change per 1%
AUX	Panto	7.07%
	ACM	6.54%
DYN	Gear box	4.19%
	Traction Motors	3.62%
TCMS	Master controller	7.13%
	MVB	8.00%
MEB	Air compressor	7.49%
TCMS	Frame	2.18%
	Wheels + Axles	2.16%

## Conclusion

This paper introduced a new approach to reliability and availability analysis of the electric train's rolling stock. The reliability model for a common type of electric train was accomplished and demonstrated all mathematical equations for reaching the reliability and availability parameters. The proposed method would eventually identify the critical components for the reliability of a train. This study contributes a comprehensive and effectively validated methodology for RCM in railway rolling stock, emphasizing cost reduction, system reliability, and the strategic prioritization of maintenance efforts. The practical application and validation of the proposed method is performed on Tabriz line 1 metro, located in the west of Iran under operation and service for more than six years.

- The obtained results showed that the critical components are AUX (Panto, ACM), DYN (Gearbox, Traction Motors), TCMS (Master controller, MVB), MEB (Air compressor, Pipe), and BOG (Frame, Wheels + Axles) and the operating company can be scheduled maintenance programs based on this finding to keep the train much safer.
- The results showed that the most critical parts of train reliability are from the bogie subsystem because all components are connected in a series. Also, the MTTR of the parts is much more significant than the other subsystem and the brake system is the most reliable subsystem in the train.
- The results of sensitivity analysis performed for the effect of changes in equipment repair rates on their sensitivity showed that MVB, air compressor, master controller, panto, and ACM equipment are more sensitive for 1% change in repair rate with an Average change by 8.00%, 7.49%, 7.13%, 7.07%, and 6.54%, respectively compared to other equipment and showed more changes.
- The proposed method would help the operation company manager schedule their program based on the reliability and availability parameters of the train, making the system much safer. By identifying critical components, decision-makers can prevent operational loss in the mainline. The presented approach also helps the maintenance manager manage spare parts and forecast critical components in train reliability.
- The significant advantage of the proposed method is that it can apply it in all types of electric trains with a bit of change in the reliability model and equations. Also, it does not need colossal computer processing power for calculation. As the maintenance cost in this industry is too much, future works can focus on proposing a different method for identifying the critical component and comparing the result with this approach for selecting optimal maintenance programs based on reliability and cost.
- Looking ahead, future research endeavors could explore alternative methods for identifying critical components and compare their results with our approach to facilitate the selection of optimal maintenance programs based on reliability and cost considerations also the next research can be focused on the quantitative analysis of cost reduction which this approach applied on the spare part management and their preventive maintenance scheduling.

## Data Availability

The datasets used and/or analysed during the current study available from the corresponding author on reasonable request.

## List of symbols

$$A$$: Availability indices
$$U$$: Unavailability indices
$f_{F}$: Failure mode steady-state frequency
$f_{S}$: Success mode steady-state frequency
$C_{X}^{Y}$: Impact of component X in the criticality of Y
$MCT$: Mean cycle time
$$MDT$$: Mean downtime
$$MUT$$: Mean uptime
$MTTF$: Mean time to failure
$MTTFF$: Mean time to the first failure
$P_{ + } \left( 0 \right)$: Initiation success mode probability
$P_{F}^{i}$: Failure mode probability caused by ith component
$P_{S}^{i}$: Success mode probability caused by ith component
$P_{F}^{{}}$: Failure mode probability
$P_{S}^{{}}$: Success mode probability
$R$: Matrix of transition rate
$R_{11}$: Success to success transition rates
$U_{k}$: Dimension k unit vector
$\lambda_{i}$: The component i failure rate
$\mu_{i}$: The component i repair rate
$\lambda_{ij}$: States i to j departure rate
$$\lambda_{S}$$: Failure rate
$$\mu_{S}$$: Repair rate
